# Genetic mapping of fiber color genes on two brown cotton cultivars in Xinjiang

**DOI:** 10.1186/2193-1801-3-480

**Published:** 2014-08-28

**Authors:** Lixiang Wang, Haifeng Liu, Xueyuan Li, Xiangwen Xiao, Xiantao Ai, Cheng Luo, Lihuang Zhu, Xiaobo Li

**Affiliations:** Key Laboratory of Chemistry of Plant Resources in Arid Regions, Xinjiang Technical Institute of Physics and Chemistry, Chinese Academy of Sciences, Urumqi, 830011 China; China Colored-Cotton (group) Co., Ltd., Urumqi, 830016 China; Economic Crop Research Institute, Xinjiang Academy of Agricultural Sciences, Urumqi, 830091 China; State Key Laboratory of Plant Genomics, Institute of Genetics and Developmental Biology, Chinese Academy of Sciences, Beijing, 100101 China; Key State Laboratory of Plant Cell & Chromosome Engineering Center of Agricultural Resources Research, Institute of Genetics and Developmental Biology, Chinese Academy of Sciences, 286 Huaizhong Road, Shijiazhuang, Hebei 050021 China

**Keywords:** Colored cotton, Genetic mapping, SSR

## Abstract

In the present study, genetic linkage analysis was carried out to map the fiber color loci *Lc*_*1*_ and *Lc*_*2*_ on two brown cotton cultivars with SSR and EST-SSR markers in the reference map by F_2_ segregation populations. The *Lc*_*1*_ locus carried by Xincaimian6 (*Gossypium hirsutum* L.) was flanked by the marker NAU2862 and NAU1043 on the long arm of Chromosome 07, with genetic distance 7.8 cM and 3.8 cM, respectively. The *Lc*_*2*_ carried by Xincaimian 5 (*Gossypium hirsutum* L.) was flanked by the marker NAU5433 and NAU2968 on the short arm of Chromosome 06, with genetic distance 4.4 cM and 7.4 cM respectively. Moreover, the marker NAU3735 and marker NAU5434 co-segregated with the *Lc*_*1*_ and the *Lc*_*2*_ locus, respectively. The results of marker association studies with these two loci provides the basic information for the final isolation of these important genes in colored cotton, and these linkage markers also could facilitate application of marker assisted selection in the future.

## Background

Naturally colored cotton, or colored cotton, appears as brown or green colored fiber during the fiber development process. Requiring no or less dying in the textile processing, colored cotton reduces the pollution to the environment; moreover, free from residual chemical toxicant, garments made from colored cotton are more comfortable, softer and healthier for human bodies (Yuan et al. 2012). However, colored cotton is generally inferior to white cotton, especially with respect to fiber quality, including shorter, weaker fiber and lower micronaire. There is typically a negative correlation between fiber color and fiber quality traits mainly due to the pleiotropic effects of fiber color genes. For example, the deeper fiber color is, the lower the fiber quality. It is a challenge for researchers and breeders to break the negative correlation between the fiber color and fiber quality. Mapping and cloning of fiber color genes should substantially give a clue to resolve that problem mentioned above.

Kohel carried out traditional genetics research on some brown cotton materials collected from all over the world and discovered that brown fiber was controlled by six loci (*Lc*_*1*_ *~ Lc*_*6*_). *Lc*_*1*_ and *Lc*_*2*_ control the brown color of lint, *Lc*_*3*_ controls dark brown, *Lc*_*4*_, *Lc*_*5*_ and *Lc*_*6*_ control the light brown. Furthermore, he assigned *Lc*_*1*_ to chromosome 7 and *Lc*_*2*_ to chromosome 6 by linkage to morphological markers (Kohel 1985). In this study, we aimed to map *Lc*_*1*_ and *Lc*_*2*_ loci to a detailed reference molecular map (Rong et al. 2004). Complete diallel cross between four white cotton cultivars (Xinluzao13, Xinluzao31, Zhongmiansuo41 and Zhongmiansuo45) (*Gossypium hirsutum* L.) and two elite brown cotton cultivars (Xincaimian5 and Xincaimian6) (*Gossypium hirsutum* L.) cultivated in Xinjiang were conducted, and sixteen populations were obtained. Furthermore, we used the mapping populations which were in accordance with the 3:1 Mendelian inheritance (χ^2^_0.05_ < 3.84, df =1) to locate the *Lc*_*1*_ and *Lc*_*2*_ genes in the cotton reference map. This research is a start point for further defining a finer location of the fiber color genes that may result in the eventual cloning of these genes by map-based and candidate gene approaches.

## Materials and methods

### Plant materials

In the summer of 2007, parental lines (Table 1) were crossed in all combinations including reciprocals. The F_1_ plants were self-pollinated to produce the F_2_ populations in the second year. In the summer of 2009, a total of sixteen F_2_ populations were grown, and field evaluation and genetic analysis were undertaken. All field experiments were carried out in Korla Breeding Base of China Colored-Cotton (Group) Co., Ltd.

**Table 1:** **The information of parental lines for constructing the F**_**2**_ **populations**

Lint phenotype	Cultivars	Fiber color	Material source
**Brown**	Xincaimian5	Dark brown	China Colored-Cotton Group
	Xincaimian6	Light brown	China Colored-Cotton Group
**White**	Xinluzao13	White	Nongqishi Agricultural Research Institute of XPCCs
	Xinluzao31	White	Xinjiang Kuitunwanshi Seed Industry
	Zhongmiansuo41	White	Cotton Research Institute of China
	Zhongmiansuo45	White	Cotton Research Institute of China

### Cotton DNA extraction

The genomic DNA from the parental cultivars or lines and their F_2_ segregating populations were extracted from the young leaves by the CTAB method (Paterson et al. 1993; Zhang et al. 2000).

### SSR-PCR analysis and genetic mapping

Sixty-five and seventy-one pairs of cotton simple sequence repeat (SSR) primers on Chromosome 06 and Chromosome 07 were chosen according to primer information published on Cotton Marker Database (CMD) (http://www.cottongen.org/data/markers). PCRs were performed using a PTC-200 (Bio-Rad, Hercules, CA, USA) thermocycler. The genotype of the white parent was scored as “1”, the brown parent and the heterozygous (F_1_) genotype were scored as “2” and “3” in the parents, F_1_ and F_2_ populations. Missing data were noted as “−”. The χ^2^ test for goodness of fit was used to assess the Mendelian dominant inheritance in the F_2_ segregating population. After eliminating the segregation distortion markers by χ^2^ test, other SSR markers were detected by linkage analysis.

The volume of PCR reaction system was 20 μl, including 2 μl of 10× PCR buffer (containing Mg^2+^), 1 μl of dNTPs (10 mM), 0.3 U of *Taq* enzyme (2 U/μl), 0.5 μl of SSR upstream primers and downstream primers (5 μM) and 1 μl of template DNA (40 ng/μl), 14.7 μl of ddH_2_O. Reaction procedures were 94°C for 4 min; 94°C for 40 s, 57°C for 45 s, 72°C for 50 s, 35 cycles; 72°C for 10 min, and the amplified products were preserved at 4°C. Amplified sample was mixed with 10 μl of loading buffer, denatured at 95°C for 10 min, the mixture was immediately transferred to ice-bath cooling for electrophoresis. Vertical slab denaturing polyacrylamide gel (7%) electrophoresis was used to separate the SSR amplified products, pre-electrophoresis at 120 V for 10 min, and 180 V constant voltage electrophoresis for 4 h. DNA fragments were detected with ethidium bromide staining.

### Data analysis

SPSS13.0 software was used to test for segregation ratio of selected markers in F_2_ segregating population and Mapmaker 3.0 software was employed to construct the genetic linkage map, and all linkage groups were determined at LOD scores ≥6. Mapping was completed using MapDraw software (Lander et al. 1987).

## Results

### Inheritance of fiber color

In each population, all plants in the F_1_ generation displayed the same phenotype as the brown parent. This result indicates that the brown fiber trait was determined by nuclear inheritance and brown was dominant over white. Segregation analysis of F_2_ segregation populations was accomplished by visual inspection of the lint color of individual plants in each population. Five F_2_ populations were consistent with a 3:1 ratio at significant level of α = 0.05 (Table 2). Therefore, these five populations were used for mapping. Totally, 65 and 71 SSR markers on Chromosome 06 and Chromosome 07 respectively were employed to screen polymorphisms among all six parental lines. Sixteen polymorphic marker loci, 9 on Chromosome 06 and 7 on Chromosome 07, were identified. Then, the polymorphic SSR markers between the two parental lines were run on the corresponding F_2_ populations and the marker genotypes were recorded. The mapping results were shown as follows.

**Table 2:** **White/brown cross combinations consisted with Mendel segregation ratio**

Parental combination		F _2_		Brown:white	***χ*** ^***2***^
P _1_(♂)	P _2_(♀)	White	Brown		
Xinluzao13	Xincaimian6	30	85	2.83:1	0.072
Xinluzao31	Xincaimian6	**27**	**82**	**3.07:1**	**0.012**
Zhongmiansuo41	Xincaimian6	**27**	**90**	**3.33:1**	**0.231**
Zhongmiansuo45	Xincaimian6	36	84	2.33:1	1.600
Xincaimian6	Xinluzao13	25	78	3.12:1	0.029
Xincaimian6	Xinluzao31	**26**	**76**	**2.92:1**	**0.013**
Xincaimian6	Zhongmiansuo41	17	100	6:1	—
Xincaimian6	Zhongmiansuo45	23	90	4:1	—
Xinluzao13	Xincaimian5	40	72	1.8:1	–
Xinluzao31	Xincaimian5	20	79	3.95:1	**--**
Zhongmiansuo41	Xincaimian5	**34**	**93**	**2.74:1**	**0.08**
Zhongmiansuo45	Xincaimian5	11	88	8:1	–
Xincaimian5	Xinluzao13	37	81	2.19:1	–
Xincaimian5	Xinluzao31	**28**	**92**	**3.32:1**	**0.223**
Xincaimian5	Zhongmiansuo41	17	100	5.88:1	—
Xincaimian5	Zhongmiansuo45	23	96	4.17:1	—

Note: *χ* ^*2*^_0.05_ = 3.84, df = 1. Bold numbers represent F2 populations which were used for linkage analysis and genetic mapping of Lc1 and Lc2.

### Genetic mapping of *Lc*_*1*_

**Figure 1:**
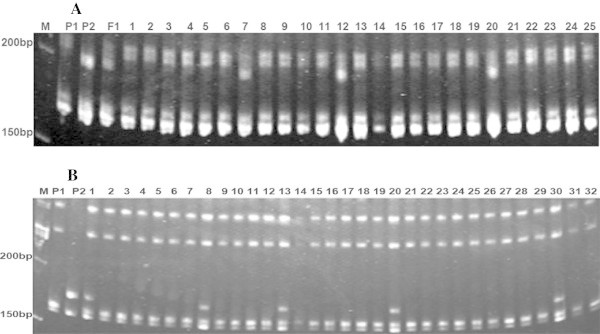
**PCR amplification products generated by SSR primer pairs in F2 segregation populations. A**. Segregation of SSR marker NAU1043 in F_2_ population from Xincaimian 6 × Xinluzao31. Lane M is a 100-bp molecular weight marker. Lanes 2, 3, and 4 are parental lines Xinluzao31, Xincaimian 6 and F_1_. Lanes 5–25 are a subset of the F_2_ individuals; **B**. Segregation of SSR marker NAU5433 in F_2_ population from Zhongmiansuo41 × Xincaimian5. Lane M is a 100-bp molecular weight marker. Lanes 2, 3, and 4 are parental lines Zhongmiansuo41, Xincaimian5 and F_1_. Lanes 5–32 are a subset of the F_2_ individuals.

**Figure 2:**
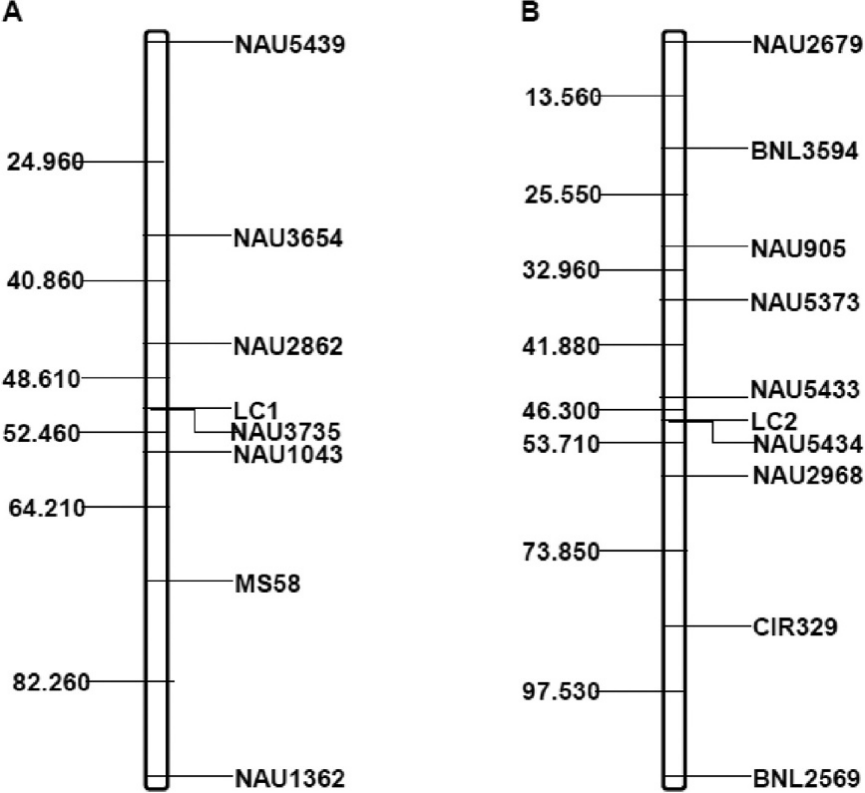
**Mapping of** ***Lc***_***1***_ **and** ***Lc***_***2***_ **. A**. Mapping of *Lc*_*1*_ gene on Chromosome 07; **B**. Mapping of *Lc*_*2*_ gene on Chromosome 06.

Linkage analysis suggested that the fiber color gene carried by Xincaimian6, *Lc*_*1*_, is preliminarily located between NAU3654 and MS58 on the long arm of Chromosome 07, based on analysis of three F_2_ populations derived from Zhongmiansuo41 × Xincaimian6, Xincaimian6 × Xinluzao31 and Xinluzao31 × Xincaimian6. To narrow down the *Lc*_*1*_ locus region further, three SSR markers, NAU2862, NAU3735 and NAU1043, were detected as DNA polymorphisms between Xincaimian6 and Xinluzao31 (Figure 1A). *Lc*_*1*_ co-segregated with the marker NAU3735, and flanked by markers NAU2862 and NAU1043, with genetic distance of 7.8 cM and 3.8 cM, respectively (Figure 2A).

### Genetic mapping of *Lc*_*2*_

Linkage analysis also suggested that the fiber color gene carried by Xincaimian5, *Lc*_*2*_, is preliminarily located between CIR329 and NAU905 in the short arm of Chromosome 06 by F_2_ populations derived from Zhongmiansuo41 × Xincaimian5 and Xincaimian5 × Xinluzao31. To narrow down the *Lc*_*2*_ locus region further, four SSR markers, NAU5373, NAU5433, NAU5434 and NAU2968, were detected as DNA polymorphisms between Zhongmiansuo41 and Xincaimian5 (Figure 1B). *Lc*_*2*_ co-segregated with the marker NAU5434, and flanked by NAU5433 and NAU2968, with genetic distance of 4.4 cM and 7.4 cM, respectively (Figure 2B).

## Discussion

The inheritance of cotton fiber color trait has been studied in several reports and a few of genetic loci potentially involved in fiber color formation have been identified (Harland 1935; Симонгулян 1984; Kohel 1985; Shi et al. 2002). Harland (1935) found that the inter-barbadense cross Egyptian brown × Sea Island white gave F1 intermediate and complicated segregation of the blending type in F2 and the factor K^B^ ( brown lint) of the brown parent was accompanied by a number of plus modifiers absent in the white parent. He also concluded that brown lint in barbadense and hirsutum was not determined by the same gene, but by a pair of duplicate genes. Симонгулян (1984) made a conclusion through the analysis of a hybrid between white lint upland cotton and brown semi-wild Mexican species (*G. hirsutum*) that the brown fiber was controlled by two pairs of complementary major genes *Lc*_*1*_ and *Lc*_*2*_, lack of any pair of dominant alleles lint became white in color, and another gene *Lc*_*3*_ was a supplementary gene which might strengthen the function of these two pairs of genes. Shi et al. (2002) concluded that brown and green colored lint were controlled by one pair of major genes incomplete in dominance on non-homologous chromosomes, and that there were genetic interactions between lint and fuzz coloring genes. Interestingly, in our studies, we noticed that some F_2_ populations fitted a 3:1 ratio regardless of cross or reverse cross, e.g. a combination of Xinluzao 31 and Xincaimian 6, but some do not, e.g. a combination of Zhongmiansuo 41 and Xincaimian 5. Therefore, we deduced that modification of minor genes resulted in the difference in proportion of F_2_ in reciprocal crosses.

It has been previously demonstrated that brown cotton varieties in Xinjiang had nearer genetic relationships with upland cotton (*G. hirsutum*), but far from Sea-island cotton *(G. barbadense)* (Wang et al. 2012). For many years, breeders have tried to improve the fiber quality of colored cotton by introgressing fiber quality traits from sea island cotton, but the results were poor (Lacape et al. 2005). For map-based cloning, because chromosome variability of target genes can be ensured between upland cotton (*G. hirsutum*) and sea island cotton (*G. barbadense*), and genetic map of both cotton species can be fully utilized as well, *G. hirsutum* × *G. barbadense* populations are usually chosen in map-based cloning (Park et al. 2005). However, in fact, few progenies of F_1_ were fertile, and severe segregation distortion was observed in F_2_ populations by brown cotton crossing various sea island cultivars. Distant hybridization-sterility between current brown cotton cultivars and Sea-island cotton cultivar is a bottleneck not only for the improvement of fiber quality but also for map-based cloning fiber color genes in colored cotton (Zhang et al. 1994).

Another of the major limitations to map-based cloning for the genes of interest in cotton is the lack of polymorphism resulting from a narrow genetic base. Ling et al. reported that genetic similarities based on Jaccard’s similarity coefficient between brown cotton and white upland cotton was an average of 0.7 using sequence-related amplified polymorphism (SRAP) markers, which suggests that these cultivars are very closely related concerning their genetic background or they have a common ancestor (Ling et al. 2009; Guo et al. 2004). In China, three major cotton growing agro-ecological zones have been divided based on cotton type, distribution and growth environment, including the northwest inland cotton region, the Yellow River valley region and Yangtze River valley region. Presently, according to the pedigrees known, brown cotton cultivars had nearer genetic relationships with native upland cotton varieties in Xinjiang than those cultivars in the other two regions. Therefore, the cotton varieties in the other two regions are preferred to choose as parental lines for constructing segregation populations; on the other hand, more markers, such as single nucleotide polymorphism (SNP), cleaved amplified polymorphic sequences (CAPS) markers, need to be developed for further fine mapping. Many studies covering physiology, biochemistry, cell genetics and conventional breeding have been reported using colored cotton as subject materials (Qiu 2004).

In conclusion, we fulfilled the primary purpose of mapping the *Lc*_*1*_ and *Lc*_*2*_ loci using SSR markers. These findings could be used for marker-assistant selection breeding. To the best of our knowledge, this is the first effort at mapping fiber color genes of brown cotton cultivars using SSR markers. Fine mapping will be further carried on by enlarging the F_2_ populations constructed by near-isogenic line and developing the new markers. With the more sequences release of tetraploid cotton, final cloning of the fiber color genes would help us to understand the complex molecular mechanism of color development in cotton fiber.

## Abbreviations

SSR: Simple sequence repeats
CMD: Cotton marker database
SRAP: Sequence-related amplified polymorphism
CAPS: Cleaved amplified polymorphic sequences.
